# The Solution of Fully Fuzzy Quadratic Equation Based on Optimization Theory

**DOI:** 10.1155/2014/156203

**Published:** 2014-06-09

**Authors:** T. Allahviranloo, L. Gerami Moazam

**Affiliations:** Department of Mathematics, Islamic Azad University, Science and Research Branch, Tehran, Iran

## Abstract

Firstly in this paper we introduce a new concept of the 2nd power of a fuzzy number. It is exponent to production (EP) method that provides an analytical and approximate solution for fully fuzzy quadratic equation (FFQE) : $F(\tilde{X})=\tilde{D}$, where $F(\tilde{X})=\tilde{A}{\tilde{X}}^{2}+\tilde{B}\tilde{X}+\tilde{C}$. To use the mentioned EP method, at first the 1-cut solution of FFQE as a real root is obtained and then unknown manipulated unsymmetrical spreads are allocated to the core point. To this purpose we find *λ* and *μ* as optimum values which construct the best spreads. Finally to illustrate easy application and rich behavior of EP method, several examples are given.

## 1. Introduction

The problem of finding the roots of equations like the quadratic equation has many applications in applied sciences like finance [1, 2], economy [3–6], and mechanics [7]. Sevastjanov and Dymova [8] proposed a new method for solving interval and fuzzy linear equations. In [9–11] Buckly discussed solving fuzzy equations. Abbasbandy and Otadi in [12] obtained the real valued roots of fuzzy polynomials using fuzzy neural networks. In [13–16] the authors have introduced numerical and neural net solutions to solve fuzzy equations. In the current paper we propose a new method to solve $\tilde{A}{\tilde{X}}^{2}+\tilde{B}\tilde{X}+\tilde{C}=\tilde{D}$ that the complication of arithmetics does not depend on $\tilde{A}$, $\tilde{B}$, and $\tilde{X}$ being positive or negative. This method solves some problems that have no analytical solution and this is an advantage of EP method because, as we know, numerical methods need initial guess to continue and the new method provides this neediness.

The rest of the paper is set out as follows. In the second section some related basic definitions of fuzzy mathematics for the analysis are recalled. In Section 3, a new method, called EP, for solving fully fuzzy quadratic equation is presented. In Section 4, this method is used for an analytical approximate solution of FFQE. In Section 5, the conclusions are drawn.

## 2. Basic Concepts

The basic definitions are given as follows.

**Definition 1 (see [17–20])**

A fuzzy number is a function $\tilde{u}:R\to [0,1]$ which satisfies the following:

1. $\tilde{u}$ is upper semicontinuous on *R*;
2. $\tilde{u}$ is normal; that is, ∃*x*_0_ ∈ *R* with $\tilde{u}(x_{0})=1$;
3. $\tilde{u}$ is convex fuzzy set;
4. $\bar{\left\{x\in R|\tilde{u}(x)>0\right\}}$ is compact, where $\overset{-}{A}$ denotes the closure of *A*.

Note: in this paper we consider fuzzy numbers which have a unique *x*_0_ ∈ *R* with $\tilde{u}(x_{0})=1$ [18].

The set of all these fuzzy numbers is denoted by *F*. Obviously, *R*⊆*F*. For 0 < *r* ≤ 1, we define *r*-cut of fuzzy number $\tilde{u}$ as ${\left[\tilde{u}\right]}_{r}=\{x\in R:\tilde{u}(x)\geq r\}$ and ${\left[\tilde{u}\right]}_{0}=\bar{\left\{x\in R:\tilde{u}(x)\geq 0\right\}}$. In [8] from (4)–(10) it follows that ${\left[\tilde{u}\right]}_{r}$ is a bounded closed interval for each *r* ∈ [0,1]. We denote the *r*-cut of fuzzy number $\tilde{u}$ as ${\left[\tilde{u}\right]}_{r}=[\underline{u}(r),\bar{u}(r)]$.

**Definition 2 (see [18])**

A fuzzy number $\tilde{u}$ is positive (negative) if $\tilde{u}(x)=0$ for all *x* < 0  (*x* > 0).

**Definition 3 (see [21, 22])**

A fuzzy number $\tilde{u}$ in parametric form is a pair $\left(\underline{u},\bar{u}\right)$ of functions $\underline{u}(r)$ and $\bar{u}(r)$, 0 ≤ *r* ≤ 1, which satisfy the following requirements:

1. $\underline{u}(r)$ is a bounded nondecreasing left continuous function in [0,1];
2. $\bar{u}(r)$ is a bounded nonincreasing left continuous function in [0,1];
3. $\underline{u}(r)\leq \bar{u}(r)$, 0 ≤ *r* ≤ 1.

**Definition 4 (see [21])**

For arbitrary $\tilde{u}=(\underline{u}(r),\bar{u}(r))$ and $\tilde{v}=(\underline{v}(r),\bar{v}(r))$, 0 ≤ *r* ≤ 1, and scalar *k*, we define addition, subtraction, and scalar product by *k* and multiplication is, respectively, as follows.

 Addition: $\underline{u+v}(r)=\underline{u}(r)+\underline{v}(r)$; $\bar{u+v}(r)=\bar{u}(r)+\bar{v}(r)$.
 Subtraction: $\underline{u-v}(r)=\underline{u}(r)-\bar{v}(r)$; $\bar{u-v}(r)=\bar{u}(r)-\underline{v}(r)$.
 Scalar product:
  (1) $$\begin{array}{c} k\tilde{u}=\{\begin{array}{cc} \left(k\underline{u}\left(r\right),k\bar{u}\left(r\right)\right), & k\geq 0\\ \left(k\bar{u}\left(r\right),k\underline{u}\left(r\right)\right), & k<0. \end{array} \end{array}$$
 Multiplication:
  (2) uv_(r)=min⁡{u_(r)v_(r),u_(r)v¯(r),u¯(r)v_(r),u¯(r)v¯(r)}uv¯(r)=max⁡{u_(r)v_(r),u_(r)v¯(r),u¯(r)v_(r),u¯(r)v¯(r)}.

For two important cases multiplication of two fuzzy numbers is defined by the following terms.

 If $\tilde{u}\geq 0$ and $\tilde{v}\geq 0$, then $\underline{uv}(r)=\underline{u}(r)\underline{v}(r)$ and $\bar{uv}(r)=\bar{u}(r)\bar{v}(r)$.
 If $\tilde{u}\leq 0$ and $\tilde{v}\leq 0$, then $\underline{uv}(r)=\bar{u}(r)\bar{v}(r)$ and $\bar{uv}(r)=\underline{u}(r)\underline{v}(r)$.
 If $\tilde{u}\geq 0$ and $\tilde{v}\leq 0$, then $\underline{uv}(r)=\bar{u}(r)\underline{v}(r)$ and $\bar{uv}(r)=\underline{u}(r)\bar{v}(r)$.
 If $\tilde{u}\leq 0$ and $\tilde{v}\geq 0$, then $\underline{uv}(r)=\underline{u}(r)\bar{v}(r)$ and $\bar{uv}(r)=\underline{u}(r)\bar{v}(r)$.

Arithmetics of *r*-cuts is similar to arithmetics of the parametric form recalled previously [23, 24].

**Definition 5 (see [21])**

Two fuzzy numbers $\tilde{u}$ and $\tilde{v}$ are said to be equal, if and only if $\underline{u}(r)=\underline{v}(r)$ and $\bar{u}(r)=\bar{v}(r)$, for each *r* ∈ [0,1].

A crisp number *α* in parametric form is $\underline{u}(r)=\bar{u}(r)=\alpha$, 0 ≤ *r* ≤ 1. A triangular fuzzy number is popular and represented by $\tilde{u}=(m,\alpha ,\beta )$, where *α* > 0 and *β* > 0, which has the parametric form as follows:

(3) $$\begin{array}{c} \underline{u}\left(r\right)=m-\alpha +r\alpha ,\;\;\bar{u}\left(r\right)=m+\beta -r\beta . \end{array}$$

**Definition 6 (see [25])**

Let *D* : *F* × *F* → *R* ∪ {0} and let $D(\tilde{u},\tilde{v})$ = $\sup_{r\in [0,1]}\max\left\{\left|\underline{u}\left(r\right)-\underline{v}\left(r\right)\right|,\left|\bar{u}\left(r\right)-\bar{v}\left(r\right)\right|\right\}$ be the Hausdorff distance between fuzzy numbers, where ${\left[\tilde{u}\right]}_{r}=[\underline{u}(r),\bar{u}(r)]$ and ${\left[\tilde{v}\right]}_{r}=[\underline{v}(r),\bar{v}(r)]$. The following properties are well known:

1. $D(\tilde{u}⨁\tilde{w},\tilde{v}⨁\tilde{w})=D(\tilde{u},\tilde{v})$, for all $\tilde{u}$, $\tilde{v}$, $\tilde{w}\in F$;
2. $D(k⨀\tilde{u},k⨀\tilde{v})=\left|k\right|D(\tilde{u},\tilde{v})$, for all *k* ∈ *R* and $\tilde{u}$, $\tilde{v}\in F$;
3. $D(\tilde{u}⨁\tilde{v},\tilde{w}⨁\tilde{e})\leq D(\tilde{u},\tilde{w})+D(\tilde{v},\tilde{e})$, for all $\tilde{u}$, $\tilde{v}$, $\tilde{w}$, $\tilde{e}\in F$.

Therefore (*F*, *D*) is a complete metric space.

## 3. Exponent to Production Method

Let $\tilde{A}=(\underline{a}(r),\bar{a}(r))$, $\tilde{B}=(\underline{b}(r),\bar{b}(r))$, $\tilde{C}=(\underline{c}(r),\bar{c}(r))$, $\tilde{D}=(\underline{d}(r),\bar{d}(r))$, $\tilde{X}=(\underline{x}(r),\bar{x}(r))$ and

(4) $$\begin{array}{c} F\left(\tilde{X}\right)=\tilde{D}, \end{array}$$

(5) $$\begin{array}{c} F\left(\tilde{X}\right)=\left(\underline{F}\left(\underline{x},\bar{x},r\right),\bar{F}\left(\underline{x},\bar{x},r\right)\right), \end{array}$$

where $F(\tilde{X})=\tilde{A}{\tilde{X}}^{2}+\tilde{B}\tilde{X}+\tilde{C}$. In this method, we convert the 2nd exponent of a fuzzy number to a product of two fuzzy numbers in parametric form. By this conversion we obtain an analytical and approximate solution for a FFQE. In EP method, at first, we find *α*, as a real root of crisp 1-cut equation and then we get $\tilde{X}=\left(\alpha -f_{1}\left(\alpha_{1}\left(r\right),\alpha_{2}\left(r\right)\right),\alpha +f_{2}\left(\alpha_{1}\left(r\right),\alpha_{2}\left(r\right)\right)\right)$ as solution of (4) and apply the approximation ${\tilde{X}}^{2}≅\left(\alpha -\alpha_{1}\left(r\right),\alpha +\alpha_{1}\left(r\right)\right)\left(\alpha -\alpha_{2}\left(r\right),\alpha +\alpha_{2}\left(r\right)\right)$, in which *α*_1_(*r*)⩾0, *α*_2_(*r*)⩾0, *α*_1_′(*r*) < 0, and *α*_2_′(*r*) < 0. To find *f*_*i*_(*α*_1_(*r*), *α*_2_(*r*)), for *i* = 1,2, we consider two cases:

1. FFQE has analytical solution;
2. FFQE does not have analytical solution.

### 3.1. Case (1)

In this case we have $\underline{x}(0)⩽\underline{x}(1)=\bar{x}(1)⩽\bar{x}(0)$. Therefore we construct conditions that provide a good approximation. These conditions are as follows:

1. *f*_*i*_(*α*_1_(1), *α*_2_(1)) = 0, for *i* = 1,2;
2. $\alpha -f_{1}\left(\alpha_{1}\left(0\right),\alpha_{2}\left(0\right)\right)=\underline{x}(0)$ and $\alpha +f_{2}\left(\alpha_{1}\left(0\right),\alpha_{2}\left(0\right)\right)=\bar{x}(0)$.

To find *f*_*i*_(*α*_1_(*r*), *α*_2_(*r*)), *i* = 1,2, at first we substitute $\tilde{X}$ = (*α* − *f*_1_(*α*_1_(*r*), *α*_2_(*r*)), *α* + *f*_2_(*α*_1_(*r*), *α*_2_(*r*))) and ${\tilde{X}}^{2}$ *≅*(*α* − *α*_1_(*r*), *α* + *α*_1_(*r*))(*α* − *α*_2_(*r*), *α* + *α*_2_(*r*)), in parametric form of (4), and then we obtain

(6) (a_(r),a¯(r))(α−α1(r),α+α1(r))(α−α2(r),α+α2(r))  +(b_(r),b¯(r))  ×(α−f1(α1(r),α2(r)),α+f2(α1(r),α2(r)))  +(c_(r),c¯(r))=(d_(r),d¯(r)).

Set *r* = 1. Using condition (1) and $\underline{a}(1)=\bar{a}(1)$, $\underline{b}(1)=\bar{b}(1)$, $\underline{c}(1)=\bar{c}(1)$, and $\underline{d}(1)=\bar{d}(1)$, we obtain

(7) $$\begin{array}{c} \alpha \left(\alpha_{1}\left(1\right)+\alpha_{2}\left(1\right)\right)=0, \end{array}$$

for each *α* ∈ *R*; that means

(8) $$\begin{array}{c} \alpha_{1}\left(1\right)+\alpha_{2}\left(1\right)=0. \end{array}$$

By this conclusion we decide to get

(9) f1(α1(r),α2(r))=λ(α1(r)+α2(r)),f2(α1(r),α2(r))=μ(α1(r)+α2(r)),λ,μ>0, λ,μ∈R.

Using condition (2) we have

(10) $$\begin{array}{c} \alpha -\lambda \left(\alpha_{1}\left(0\right)+\alpha_{2}\left(0\right)\right)=\underline{x}\left(0\right),\\ \alpha +\mu \left(\alpha_{1}\left(0\right)+\alpha_{2}\left(0\right)\right)=\bar{x}\left(0\right). \end{array}$$

Up to now we have two equations and three unknown *λ*, *μ*, and *χ*(0) = *α*_1_(0) + *α*_2_(0). The third equation comes from the equality below, for *r* = 0,

(11) (a_(r),a¯(r))(α−α1(r),α+α1(r))(α−α2(r),α+α2(r))  +(b_(r),b¯(r))(α−λ(α1+α2)(r),α+μ(α1+α2)(r))  +(c_(r),c¯(r))=(d_(r),d¯(r)).

Now we can find *λ*, *μ*, and *χ*(0). To construct solution of the new method, in the above parametric form, let *χ*(*r*) = *α*_1_(*r*) + *α*_2_(*r*) and *ν*(*r*) = *α*_1_(*r*) × *α*_2_(*r*); then, by solving a 2 × 2 system via *χ*(*r*) and *ν*(*r*), we obtain *χ*(*r*) and we set

(12) $$\begin{array}{c} \tilde{X}=\left(\alpha -\lambda \chi \left(r\right),\alpha +\mu \chi \left(r\right)\right) \end{array}$$

as a solution of (4). Notice that always we have real spreads, which means *α*_1_(*r*) + *α*_2_(*r*) ∈ *R*, because *α*_1_(*r*) and *α*_2_(*r*) are the roots of *Z* ^2^ − *χ*(*r*)*Z* + *ν*(*r*) = 0.

Using the proposed method we obtain the following set of expressions for *χ*(*r*).

*Case (1).* $\tilde{X}>0:$

(1) if $\tilde{A}>0$ and $\tilde{B}<0$, then
  (13) $$\begin{array}{c} \chi \left(r\right)=\frac{\bar{a}\left(r\right)\underline{G}\left(\alpha ,\alpha ,r\right)-\underline{a}\left(r\right)\bar{G}\left(\alpha ,\alpha ,r\right)}{\underline{a}\left(r\right)UU^{-}\left(\lambda ,r\right)+\bar{a}\left(r\right)LL^{-}\left(\mu ,r\right)}; \end{array}$$
(2) if $\tilde{A}>0$ and $\tilde{B}>0$, then
  (14) $$\begin{array}{c} \chi \left(r\right)=\frac{\bar{a}\left(r\right)\underline{G}\left(\alpha ,\alpha ,r\right)-\underline{a}\left(r\right)\bar{G}\left(\alpha ,\alpha ,r\right)}{\underline{a}\left(r\right)UU^{+}\left(\mu ,r\right)+\bar{a}\left(r\right)LL^{+}\left(\lambda ,r\right)}; \end{array}$$
(3) if $\tilde{A}<0$ and $\tilde{B}>0$, then
  (15) $$\begin{array}{c} \chi \left(r\right)=-\frac{\bar{a}\left(r\right)\underline{G}\left(\alpha ,\alpha ,r\right)-\underline{a}\left(r\right)\bar{G}\left(\alpha ,\alpha ,r\right)}{\underline{a}\left(r\right)UU^{-}\left(\mu ,r\right)+\bar{a}\left(r\right)LL^{-}\left(\lambda ,r\right)}; \end{array}$$
(4) if $\tilde{A}<0$ and $\tilde{B}<0$, then
  (16) $$\begin{array}{c} \chi \left(r\right)=-\frac{\bar{a}\left(r\right)\underline{G}\left(\alpha ,\alpha ,r\right)-\underline{a}\left(r\right)\bar{G}\left(\alpha ,\alpha ,r\right)}{\underline{a}\left(r\right)UU^{+}\left(\lambda ,r\right)+\bar{a}\left(r\right)LL^{+}\left(\mu ,r\right)}. \end{array}$$

*Case (2).* $\tilde{X}<0:$

(1) if $\tilde{A}>0$ and $\tilde{B}<0$, then
  (17) $$\begin{array}{c} \chi \left(r\right)=-\frac{\bar{a}\left(r\right)\underline{G}\left(\alpha ,\alpha ,r\right)-\underline{a}\left(r\right)\bar{G}\left(\alpha ,\alpha ,r\right)}{\underline{a}\left(r\right)UL^{+}\left(\lambda ,r\right)+\bar{a}\left(r\right)LU^{+}\left(\mu ,r\right)}; \end{array}$$
(2) if $\tilde{A}>0$ and $\tilde{B}>0$, then
  (18) $$\begin{array}{c} \chi \left(r\right)=-\frac{\bar{a}\left(r\right)\underline{G}\left(\alpha ,\alpha ,r\right)-\underline{a}\left(r\right)\bar{G}\left(\alpha ,\alpha ,r\right)}{\underline{a}\left(r\right)UL^{-}\left(\mu ,r\right)+\bar{a}\left(r\right)LU^{-}\left(\lambda ,r\right)}; \end{array}$$
(3) if $\tilde{A}<0$ and $\tilde{B}>0$, then
  (19) $$\begin{array}{c} \chi \left(r\right)=\frac{\bar{a}\left(r\right)\underline{G}\left(\alpha ,\alpha ,r\right)-\underline{a}\left(r\right)\bar{G}\left(\alpha ,\alpha ,r\right)}{\underline{a}\left(r\right)UL^{+}\left(\mu ,r\right)+\bar{a}\left(r\right)LU^{+}\left(\lambda ,r\right)}; \end{array}$$
(4) if $\tilde{A}<0$ and $\tilde{B}<0$, then
  (20) $$\begin{array}{c} \chi \left(r\right)=\frac{\bar{a}\left(r\right)\underline{G}\left(\alpha ,\alpha ,r\right)-\underline{a}\left(r\right)\bar{G}\left(\alpha ,\alpha ,r\right)}{\underline{a}\left(r\right)UL^{-}\left(\lambda ,r\right)+\bar{a}\left(r\right)LU^{-}\left(\mu ,r\right)}; \end{array}$$

where

(21) G_(x_,x¯,r)=F_(x_,x¯,r)−d_(r),G¯(x_,x¯,r)=F¯(x_,x¯,r)−d¯(r),UU†(ξ,r)=αa¯(r)†ξb¯(r),LL†(ξ,r)=αa_(r)†ξb_(r),UL†(ξ,r)=αa¯(r)†ξb_(r),LU†(ξ,r)=αa_(r)†ξb¯(r),

in which †∈{+, −} and *ξ* ∈ {*λ*, *μ*}.

### 3.2. Case (2)

In this case we do not have analytical solution, and we do not have $\underline{x}(0)$ and $\bar{x}(0)$ in which $\underline{x}(0)⩽\underline{x}(1)=\bar{x}(1)⩽\bar{x}(0)$; therefore we propose that *λ* = *μ* = 0.5 because of Lemma 7:

**Lemma 7**

If *α* is real core point of $F(\tilde{X})=\tilde{D}$ and we do not have analytical solution, then *λ* = *μ* = 0.5 is the best choice in EP method.

**Proof**

Without loss of generality suppose that $\tilde{X}>0$. Let

(22) Y~Z~=(α−α1(r),α+α1(r))(α−α2(r),α+α2(r)),X~=(α−λ(α1(r)+α2(r)),α+μ(α1(r)+α2(r))).

In EP method we use the approximation ${\tilde{X}}^{2}≅\tilde{Y}\tilde{Z}$.

To find the optimum parameters *λ* and *μ*, we must solve the minimization problem as follows:

(23) Min⁡ D(X~2,Y~Z~)st. λ,μ∈R,λ>0, μ>0,

where $D({\tilde{X}}^{2},\tilde{Y}\tilde{Z})=\sup_{r\in [0,1]}\max\{\left|\alpha \left(2\lambda -1\right)\chi \left(r\right)+\nu \left(r\right)-\lambda^{2}\chi^{2}\left(r\right)\right|,\left|\alpha \left(1-2\mu \right)\chi \left(r\right)+\nu \left(r\right)-\mu^{2}\chi^{2}\left(r\right)\right|\}$.

To delimitate maximum error for any *α* ∈ *R*, we choose *λ* = *μ* = 0.5. This completes the proof.

**Lemma 8**

Necessary condition for existence of EP solution with *λ* = *μ* = 0.5 is

(24) $$\begin{array}{c} \bar{a}\left(0\right)\underline{G}\left(\alpha ,\alpha ,0\right)-\underline{a}\left(0\right)\bar{G}\left(\alpha ,\alpha ,0\right)\geq 0,\;if\;\;\tilde{A}>0, \end{array}$$

(25) $$\begin{array}{c} \bar{a}\left(0\right)\underline{G}\left(\alpha ,\alpha ,0\right)-\underline{a}\left(0\right)\bar{G}\left(\alpha ,\alpha ,0\right)\leq 0,\;if\;\;\tilde{A}<0. \end{array}$$

**Proof**

Since *χ*(*r*) is sum of *α*_1_(*r*) and *α*_2_(*r*) and we want *α*_1_(*r*) and *α*_2_(*r*) to be nonnegative, then necessary condition for existence of EP solution with *λ* = *μ* = 0.5 is *χ*(*r*) ≥ 0 and since [*α* − 0.5*χ*(*r*), *α* + 0.5*χ*(*r*)]⊆[*α* − 0.5*χ*(0), *α* + 0.5*χ*(0)], for all 0 ≤ *r* ≤ 1, it is sufficient to have *χ*(0) ≥ 0. Considering denominators of (13)–(20) we find that (24) and (25) hold if *χ*(0) ≥ 0, and this completes the proof.

**Lemma 9**

The EP method with *λ* = *μ* = 0.5 does not have solution,

(26) $$\begin{array}{c} if\;\;\bar{a}\left(0\right)\underline{G}\left(\alpha ,\alpha ,0\right)-\underline{a}\left(0\right)\bar{G}\left(\alpha ,\alpha ,0\right)<0,\;when\;\;\tilde{A}>0, \end{array}$$

(27) $$\begin{array}{c} if\;\;\bar{a}\left(0\right)\underline{G}\left(\alpha ,\alpha ,0\right)-\underline{a}\left(0\right)\bar{G}\left(\alpha ,\alpha ,0\right)>0,\;when\;\;\tilde{A}<0. \end{array}$$

**Proof**

This lemma is conclusion of Lemma 8.

**Lemma 10**

Suppose *λ* = *μ* = 0.5 in EP method and *χ*(0) ≥ 0, then sufficient condition for existence of solution is *ν*(0) ≥ 0.

**Proof**

We know *α*_1_(*r*) and *α*_2_(*r*) are nonnegative if *χ*(*r*) ≥ 0 and *ν*(*r*) ≥ 0.

Because of construction of EP method for *λ* = *μ* = 0.5 and Lemma 8, it is obvious that we must have *ν*(0) ≥ 0.

**Lemma 11**

Suppose *χ*(0) ≥ 0 and *λ* = *μ* = 0.5 in EP method, then we have solution with $\tilde{X}>0$ as follows:

(1) if $\tilde{A}>0$ and $\tilde{B}<0$, then
  (28) $$\begin{array}{c} \underline{G}\left(\alpha ,\alpha ,0\right)-\bar{G}\left(\alpha ,\alpha ,0\right)\geq \left[UU^{-}\left(0.5,0\right)+LL^{-}\left(0.5,0\right)\right]\chi \left(0\right); \end{array}$$
(2) if $\tilde{A}>0$ and $\tilde{B}>0$, then
  (29) $$\begin{array}{c} \underline{G}\left(\alpha ,\alpha ,0\right)-\bar{G}\left(\alpha ,\alpha ,0\right)\geq \left[UU^{+}\left(0.5,0\right)+LL^{+}\left(0.5,0\right)\right]\chi \left(0\right); \end{array}$$
(3) if $\tilde{A}<0$ and $\tilde{B}>0$, then
  (30) G_(α,α,0)−G¯(α,α,0)  ≥−[UU−(0.5,0)+LL−(0.5,0)]χ(0);
(4) if $\tilde{A}<0$ and $\tilde{B}<0$, then
  (31) G_(α,α,0)−G¯(α,α,0)  ≥−[UU+(0.5,0)+LL+(0.5,0)]χ(0),

with  $\tilde{X}<0$;

(1) if $\tilde{A}>0$ and $\tilde{B}<0$, then
  (32) G_(α,α,0)−G¯(α,α,0)  ≥−[LU+(0.5,0)+UL+(0.5,0)]χ(0);
(2) if $\tilde{A}>0$ and $\tilde{B}>0$, then
  (33) G_(α,α,0)−G¯(α,α,0)  ≥−[LU−(0.5,0)+UL−(0.5,0)]χ(0);
(3) if $\tilde{A}<0$ and $\tilde{B}>0$, then
  (34) $$\begin{array}{c} \underline{G}\left(\alpha ,\alpha ,0\right)-\bar{G}\left(\alpha ,\alpha ,0\right)\geq \left[LU^{+}\left(0.5,0\right)+UL^{+}\left(0.5,0\right)\right]\chi \left(0\right); \end{array}$$
(4) if $\tilde{A}<0$ and $\tilde{B}<0$, then
  (35) $$\begin{array}{c} \underline{G}\left(\alpha ,\alpha ,0\right)-\bar{G}\left(\alpha ,\alpha ,0\right)\geq \left[LU^{-}\left(0.5,0\right)+UL^{-}\left(0.5,0\right)\right]\chi \left(0\right). \end{array}$$

**Proof**

We consider only one case to discuss. We consider $\tilde{A}>0$, $\tilde{B}<0$, and $\tilde{X}>0$ and solve (11) via *χ*(*r*) and *ν*(*r*) in *r* = 0; we obtain

(36) ν(0)=(G_(α,α,0)−G¯(α,α,0) −(UU−(0.5,0)+LL−(0.5,0))χ(0)) ×[a¯(0)−a_(0)]−1.

This is obvious that *ν*(0) ≥ 0, if the numerator is nonnegative and this completes the proof.

## 4. Numerical Examples

In the next examples we use round numbers with approximation less than 10^−4^.

**Example 1**

Let $\tilde{A}=(4/1/1)$, $\tilde{B}=(2/1/1)$, $\tilde{C}=(1/1/1)$, and $\tilde{D}=(3/2/2)$ [9].

We will look for a solution where $\tilde{X}\geq 0$.

Equation $F(\tilde{X})=\tilde{D}$ becomes in parametric form as follows:

(37) (3+r,5−r)(x_2(r),x¯2(r))+(1+r,3−r)(x_(r),x¯(r))  +(r,2−r)=(1+2r,5−2r).

 The real roots of 1-cut and 0-cut equations are $\alpha =\underline{x}(1)=\bar{x}(1)=0.5$, $\underline{x}(0)=0.4343$, and $\bar{x}(0)=0.5307$.

Therefore we have analytical solution and EP solution by (24) and (29).

By (10) and (14), we find *λ* = 0.7067, *μ* = 0.3296, and

(38) $$\begin{array}{c} \chi \left(r\right)=\frac{2-2r}{-r^{2}+2r+15+\mu \left(9-r^{2}\right)-\lambda \left(r^{2}-4r-5\right)}. \end{array}$$

In this example, with $\tilde{X}=(0.5-\lambda \chi (r),0.5+\mu \chi (r))$, Hausdorff metric is $D(F(\tilde{X}),\tilde{D})=0.0228$.

**Example 2**

Letting $\tilde{A}=(4/2/2)$, $\tilde{B}=(2/2/2)$, and $\tilde{D}=(1/0.5/0.5)$, we have

(39) (2+2r,6−2r)(x_2(r),x¯2(r))+(2r,4−2r)(x_(r),x¯(r))  =(0.5+0.5r,1.5−0.5r).

The real roots of 1-cut and 0-cut equations are $\alpha =\underline{x}(1)=\bar{x}(1)=\left(-1+\sqrt{5}\right)/4=0.3090$, $\underline{x}(0)=0.5$, and $\bar{x}(0)=0.2676$.

Therefore this example does not have analytical solution. We look for EP solution. By (26) we find that this example does not have EP solution with *λ* = *μ* = 0.5 too.

Now we consider an example with $\tilde{A}<0$, $\tilde{B}>0$, and $\tilde{X}>0$.

**Example 3**

Letting $\tilde{A}=(-2/1/1)$, $\tilde{B}=(3/1/2)$, and $\tilde{D}=(1/1/2)$, we have

(40) (−3+r,−1−r)(x_2(r),x¯2(r))+(2+r,5−2r)(x_(r),x¯(r))  =(r,3−2r).

The real roots of 1-cut and 0-cut equations are $\alpha =\underline{x}(1)=\bar{x}(1)=0.5,1$, $\underline{x}(0)=0.7838$, 1.2527, and $\bar{x}(0)=0.7229$, 0.914.

Therefore, this example does not have analytical solution. We look for EP solution. By (25), (30), and (15), we find that this example has EP solution with *λ* = *μ* = 0.5 as follows: $\tilde{X}=(0.5-0.5\chi (r),0.5+0.5\chi (r))$ that

(41) $$\begin{array}{c} \chi \left(r\right)=\frac{-r^{2}+6r-5}{-r^{2}+4r-23}, \end{array}$$

and 0-cut of EP solution is $\underline{x}(0)=0.3913$ and $\bar{x}(0)=0.6087$. In this example by using Hausdorff metric we have $D(F(\tilde{X}),\tilde{D})=0.3290$.

Notice that the numerical methods needed $\underline{x}(0)$ and $\bar{x}(0)$ to obtain initial guess and often these values achieve analytical solution, but in Example 3 these values achieve EP method because in this example we do not have analytical solution.

## 5. Conclusion

In this paper we introduced a new method to solve a fully fuzzy quadratic equation. To this purpose we found the optimum spreads to decrease maximum error. One of the advantages of this method is that complications do not depend on the sign of the coefficients and variable. It is possible that these equations do not have any analytical solution, but the proposed method gives us an approximate analytical solution.

## References

[1] Buckley JJ (1987). The fuzzy mathematics of finance. *Fuzzy Sets and Systems*.

[2] Li Calzi M (1990). Towards a general setting for the fuzzy mathematics of finance. *Fuzzy Sets and Systems*.

[3] Buckley JJ (1992). Solving fuzzy equations in economics and finance. *Fuzzy Sets and Systems*.

[4] Buckley JJ, Qu Y (1991). Solving systems of linear fuzzy equations. *Fuzzy Sets and Systems*.

[5] Li Q-X, Liu S-F (2008). The foundation of the grey matrix and the grey input-output analysis. *Applied Mathematical Modelling*.

[6] Wu CC, Chang NB (2003). Grey input-output analysis and its application for environmental cost allocation. *European Journal of Operational Research*.

[7] Chen S-H, Yang X-W (2000). Interval finite element method for beam structures. *Finite Elements in Analysis and Design*.

[8] Sevastjanov P, Dymova L (2009). A new method for solving interval and fuzzy equations: linear case. *Information Sciences*.

[9] Buckley JJ, Qu Y (1990). Solving linear and quadratic fuzzy equations. *Fuzzy Sets and Systems*.

[10] Buckley JJ, Qu Y (1990). On using *α*-cuts to evaluate fuzzy equations. *Fuzzy Sets and Systems*.

[11] Buckley JJ, Qu Y (1991). Solving fuzzy equations: a new solution concept. *Fuzzy Sets and Systems*.

[12] Abbasbandy S, Otadi M (2006). Numerical solution of fuzzy polynomials by fuzzy neural network. *Applied Mathematics and Computation*.

[13] Abbasbandy S, Asady B (2004). Newton’s method for solving fuzzy nonlinear equations. *Applied Mathematics and Computation*.

[14] Allahviranloo T, Otadi M, Mosleh M (2008). Iterative method for fuzzy equations. *Soft Computing*.

[15] Buckley JJ, Eslami E (1997). Neural net solutions to fuzzy problems: the quadratic equation. *Fuzzy Sets and Systems*.

[16] Buckley JJ, Eslami E, Hayashi Y (1997). Solving fuzzy equations using neural nets. *Fuzzy Sets and Systems*.

[17] Dubois D, Prade H (1978). Operations on fuzzy numbers. *Journal of Systems Sience*.

[18] Dubois D, Prade H (1980). *Theory and Application, Fuzzy Sets and Systems*.

[19] Zadeh LA (1965). Fuzzy sets. *Information and Control*.

[20] Zimmermann HJ (1991). *Fuzzy Set Theory and Its Applications*.

[21] Cong-Xin W, Ming M (1991). Embedding problem of fuzzy number space: part I. *Fuzzy Sets and Systems*.

[22] Goetschel R, Voxman W (1986). Elementary fuzzy calculus. *Fuzzy Sets and Systems*.

[23] Jaulin L, Kieffir M, Didrit O, Walter E (2001). *Applied Interval Analysis*.

[24] Moore RE (1966). *Interval Analysis*.

[25] Diamond P, Kloeden P (1994). *Metric Space of Fuzzy Sets*.

